# Editorial: New and emerging lipid-lowering therapies for reducing cardiovascular risk: beyond statins

**DOI:** 10.3389/fcvm.2024.1364170

**Published:** 2024-01-15

**Authors:** Fei Luo, Liqing Yu, Xunde Xian, Bo Shan, Avash Das

**Affiliations:** ^1^ Department of Cardiovascular Medicine, The Second Xiangya Hospital, Central South University, Changsha, Hunan, China; ^2^ School of Medicine, University of Maryland, Baltimore, MD, United States; ^3^ Institute of Cardiovascular Sciences and Key Laboratory of Molecular Cardiovascular Sciences, Ministry of Education, Peking University, Beijing, China; ^4^ Zhejiang Provincial Key Laboratory of Pancreatic Disease, The First Affiliated Hospital, and Institute of Translational Medicine, Zhejiang University School of Medicine, Hangzhou, China; ^5^ Department of Pathology, Beth Israel Deaconess Medical Center, Harvard Medical School, Boston, MA, United States

**Keywords:** ASCVD, LDL-C, lipid-lowering therapies, cardiovascular risk, PCSK9

**Editorial on the Research Topic** New and emerging lipid-lowering therapies for reducing cardiovascular risk: beyond statins

Atherosclerotic cardiovascular disease (ASCVD) continues to pose a significant global health challenge, contributing to high rates of morbidity and mortality. Extensive research has underscored the pivotal role of dyslipidemia, particularly the abnormal elevation of plasma low-density lipoprotein cholesterol (LDL-C) and triglycerides (TG), in the development and progression of ASCVD (1, 2). While statins have long been established as the primary pharmacological intervention for ASCVD prevention and treatment, there remains a pressing need for more effective strategies, particularly in addressing the persistent challenge of reducing TG levels.

Despite the success of statins in lowering LDL-C, a substantial residual cardiovascular risk persists among patients who adhere to statin therapy (3). Even with the advent of PCSK9 inhibitors (4), which enhance the clearance of LDL-C by up-regulating hepatic LDL receptor (LDLR), there exists a treatment gap for individuals with familial hypercholesterolemia, especially those with complete LDLR deficiency (5, 6). This unmet need presents a formidable therapeutic hurdle for lipidologists, cardiologists, and the pharmaceutical industry.

In response to this critical gap, researchers have delved into diverse therapeutic targets for lipid modulation, exploring avenues beyond LDLR-focused interventions. Pre-clinical and clinical investigations have shed light on potential targets such as angiopoietin-like protein 3 (ANGPTL3) (7, 8), apolipoprotein C3 (ApoC3) (9), apolipoprotein B (ApoB) (10), lipoprotein (a), and microsomal triglyceride transfer protein (MTTP) (11). Among these, ANGPTL3 has emerged as a particularly promising target, demonstrating the ability to effectively reduce plasma LDL-C and TG levels, independent of LDLR, in patients with refractory hypercholesterolemia, including those with heterozygous familial hypercholesterolemia (HeFH) (7, 12, 13).

The breakthrough in lipids-lowering strategies not only holds promise for addressing the limitations of current therapies but also underscores the importance of furthering our understanding of the molecular mechanisms governing lipid metabolism. It is imperative to foster a collaborative platform for robust discussion, knowledge exchange, and the dissemination of new insights into lipid metabolism regulation and emerging lipid-lowering therapies.

In this context, we organized a research topic titled “New and Emerging Lipid-Lowering Therapies for Reducing Cardiovascular Risk: Beyond Statins” for the submission of research articles, reviews, case reports, editorials, and methodology papers that investigate the intricate role of lipid metabolism in ASCVD and explore novel lipid-lowering targets. By fostering a collective effort to advance our understanding of lipid modulation and its implications for ASCVD management, we aim to catalyze the development of innovative therapeutic approaches that hold the potential to transform clinical practice and improve patient outcomes.

This issue's research topic features a total of 5 articles. The groundbreaking research by Sviridov et al., recently published in our research topic. This study introduces a novel class of short hydrocarbon stapled peptides designed to mimic the function of apolipoprotein C-II (ApoC2) and activate lipoprotein lipase, leading to a reduction in plasma triglyceride levels in mice. The findings from this study are particularly promising, as the newly developed peptides demonstrated a significant reduction in both plasma triglyceride and cholesterol levels in mice following administration. This discovery opens up new avenues for the development of innovative therapies targeting hypertriglyceridemia. While the results are compelling, the study also highlights the need for further investigation into the pharmacokinetics and pharmacodynamics of these peptides. Additionally, the authors emphasize the necessity for continued formulation work to explore the potential for oral administration of these peptides. In conclusion, this research represents a significant advancement in the field of lipid metabolism regulation, offering a potential solution for addressing hypertriglyceridemia. We eagerly anticipate future studies to build upon these findings and explore the clinical implications of these novel peptides.

The recent publication “High residual cardiovascular risk after lipid-lowering: prime time for Predictive, Preventive, Personalized, Participatory, and Psycho-cognitive medicine” by Reijnders et al. presents a compelling analysis of the persistent challenge of residual cardiovascular risk following lipid-lowering interventions. The authors advocate for a paradigm shift towards personalized medicine, emphasizing Predictive, Preventive, Personalized, Participatory, and Psycho-cognitive approaches (P5 medicine) as the optimal course of action at both population and individual levels. The review underscores the limitations of the current “one-size-fits-all” approach to healthcare and highlights the need for biologically meaningful biomarkers to accurately assess an individual's cardiovascular risk. The authors emphasize the importance of integrating diverse healthcare professionals, laboratory specialists, and innovative diagnostic approaches to address residual cardiovascular risk effectively. Furthermore, the transition from passive patient to engaged stakeholder is emphasized, promoting active patient involvement in clinical decision-making regarding their health. The authors' call for a personalized medicine strategy is not only timely but essential for optimizing patient care and outcomes. This insightful review serves as a catalyst for the integration of diverse healthcare professionals, laboratory specialists, and innovative diagnostic approaches to address residual cardiovascular risk.

The article “Genetic association of lipids and lipid-lowering drugs with sepsis: a Mendelian randomization and mediation analysis” by Lou et al., delves into the intricate genetic connections between lipids, lipid-lowering medications, and the risk of sepsis, employing innovative Mendelian randomization and mediation analysis. The study's findings shed light on the protective effects of ApoA-I and HDL-C against sepsis, while also revealing the therapeutic potential of HMGCR and CETP inhibitors beyond their lipid-lowering effects. The authors' exploration of the indirect effects of mediators, such as BMI and ApoA-I, in the treatment of sepsis with lipid-lowering drugs, offers valuable insights into the complex interplay of genetic factors in sepsis susceptibility and treatment response. This research not only enhances our understanding of the impact of lipids on sepsis patients but also paves the way for the development of novel therapeutic strategies.

“Current status and time trends of lipid and use of statins among older adults in China” (Jiang et al.) provides crucial insights into lipid levels and statin use in China's older population. The findings underscore the pressing need for improved lipid management strategies, as elevated serum lipid levels and dyslipidemia prevalence were observed among older adults. Despite an increasing trend in statin use, the achievement of treatment goals showed concerning fluctuations. The study's identification of key factors associated with statin use offers valuable guidance for healthcare practitioners and policymakers. These findings emphasize the urgency of enhancing lipid management practices to reduce the burden of cardiovascular disease in China's aging population. We look forward to further research and initiatives aimed at refining lipid management strategies and improving cardiovascular health for older adults in China.

The case report “Coronary atherosclerosis in a patient with long-standing very low LDL-C without lipid-lowering therapy” (Mottola et al.) presents a thought-provoking scenario. Despite consistently low LDL-C levels over 16 years and the absence of lipid-lowering therapy, a 60-year-old patient exhibited nonobstructive coronary atherosclerosis, challenging existing paradigms. This case underscores the intricate nature of atherosclerosis and the need for comprehensive risk factor management. It prompts a reevaluation of our understanding of atherosclerosis in the context of low LDL-C levels, emphasizing the significance of primordial prevention strategies. We anticipate that this report will stimulate further research and discussions, driving advancements in preventive interventions for atherosclerosis.

As we navigate the frontier of lipid-lowering therapies, let us embrace a spirit of collaboration and inquiry, working together to pave the way for a new era in the management of ASCVD.

## References

[1] Mach F Baigent C Catapano AL Koskinas KC Casula M Badimon L 2019 ESC/EAS guidelines for the management of dyslipidaemias: lipid modification to reduce cardiovascular risk: the task force for the management of dyslipidaemias of the European society of cardiology (ESC) and European atherosclerosis society (EAS). Eur Heart J. (2019) 41(1):111–88. 10.1093/eurheartj/ehz455 31504418

[2] Grundy SM Stone NJ Bailey AL Beam C Birtcher KK Blumenthal RS 2018 AHA/ACC/AACVPR/AAPA/ABC/ACPM/ADA/AGS/APhA/ASPC/NL-A/PCNA guideline on the management of blood cholesterol: a report of the American college of cardiology/American heart association task force on clinical practice guidelines. Circulation. (2019) 139(25):e1082–143. 10.1161/CIR.0000000000000624 30586774 PMC7403606

[3] Ferrari R Catapano AL. Residual cardiovascular risk. Eur Heart J Suppl. (2016) 18(Suppl C):C1. 10.1093/eurheartj/suw010 28533704

[4] Luo Q Tang Z Wu P Chen Z Fang Z Luo F. A bibliometric analysis of PCSK9 inhibitors from 2007 to 2022. Front Endocrinol (Lausanne). (2023) 14:1218968. 10.3389/fendo.2023.1218968 38093957 PMC10716461

[5] Raal F Durst R Bi R Talloczy Z Maheux P Lesogor A Efficacy, safety, and tolerability of inclisiran in patients with homozygous familial hypercholesterolemia: results from the ORION-5 randomized clinical trial. Circulation. (2023). 10.1161/CIRCULATIONAHA.122.063460. [Epub ahead of print] 37850379 PMC10815002

[6] Choi D Malick WA Koenig W Rader DJ Rosenson RS. Familial hypercholesterolemia: challenges for a high-risk population: JACC focus seminar 1/3. J Am Coll Cardiol. (2023) 81(16):1621–32. 10.1016/j.jacc.2023.02.038 37076217

[7] Luo F Das A Khetarpal SA Fang Z Zelniker TA Rosenson RS ANGPTL3 Inhibition, dyslipidemia, and cardiovascular diseases. Trends Cardiovasc Med. (2023). 10.1016/j.tcm.2023.01.008. [Epub ahead of print] 36746257

[8] Luo Q Chen J Su Y Wu P Wang J Fang Z Correlation between serum soluble ASGR1 concentration and low-density lipoprotein cholesterol levels: a cross-sectional study. Lipids Health Dis. (2023) 22(1):142. 10.1186/s12944-023-01910-3 37667265 PMC10476293

[9] Gaudet D Alexander VJ Baker BF Brisson D Tremblay K Singleton W Antisense inhibition of apolipoprotein C-III in patients with hypertriglyceridemia. N Engl J Med. (2015) 373(5):438–47. 10.1056/NEJMoa1400283 26222559

[10] Nurmohamed NS Navar AM Kastelein JJP. New and emerging therapies for reduction of LDL-cholesterol and apolipoprotein B: JACC focus seminar 1/4. J Am Coll Cardiol. (2021) 77(12):1564–75. 10.1016/j.jacc.2020.11.079 33766264

[11] Cuchel M Meagher EA du Toit Theron H Blom DJ Marais AD Hegele RA Efficacy and safety of a microsomal triglyceride transfer protein inhibitor in patients with homozygous familial hypercholesterolaemia: a single-arm, open-label, phase 3 study. Lancet. (2013) 381(9860):40–6. 10.1016/S0140-6736(12)61731-0 23122768 PMC4587657

[12] Luo F Das A Fang Z. Evinacumab for homozygous familial hypercholesterolemia. N Engl J Med. (2021) 384(6):e17. 10.1056/NEJMc2033612 33567206

[13] Raal FJ Rosenson RS Reeskamp LF Hovingh GK Kastelein JJP Rubba P Evinacumab for homozygous familial Hypercholesterolemia. N Engl J Med. (2020) 383(8):711–20. 10.1056/NEJMoa2004215 32813947

